# Supplementary material for: A comparative study of causal perception in Guinea baboons (Papio papio) and human adults
Source: PLoS One. 2024 Dec 12;19(12):e0311294. doi: 10.1371/journal.pone.0311294 (PMC11637404; doi:10.1371/journal.pone.0311294)
Supplement: S1 File — This file contains all supporting information including ‘S1 Table. Baboon details.’, ‘S2 Tables. Stimuli details.’ and ‘S3 Table. Categorisation strategies.’. (DOCX) [file pone.0311294.s001.docx]

# Supporting information

**S1 Table. Baboon details.** The following table shows details on the baboons participating in our experiment. Part refers to the first and second period in which we subjected the participants to the Michottean stimuli (within-subjects design). Red cells indicate that the participant did not reach the learning criteria. Green cells indicate that the participant reached the learning criteria and the number refers to the number of blocks of 60 trials needed to.

| **Name** | **Sex** | **Date of birth** | **Experiment** | | | | | | |
| --- | --- | --- | --- | --- | --- | --- | --- | --- | --- |
|  |  |  | **Part** | **Phase 1a** | **Phase 1b** | **Phase 2** | **Phase 3** | | **Phase 4** |
| **Angele** | F | 14-05-05 | 1 |  |  |  |  |  |  |
|  |  |  | 2 |  |  |  |  |  |  |
| **Arielle** | F | 25-10-05 | 1 | 24 | 11 |  | Cvs.NC | 9 |  |
|  |  |  | 2 | 107 | 50 |  | Control |  |  |
| **Articho** | M | 20-10-05 | 1 |  |  |  |  |  |  |
|  |  |  | 2 |  |  |  |  |  |  |
| **Atmosphere** | F | 08-03-98 | 1 |  |  |  |  |  |  |
|  |  |  | 2 |  |  |  |  |  |  |
| **Bobo** | M | 09-08-06 | 1 |  |  |  |  |  |  |
|  |  |  | 2 |  |  |  |  |  |  |
| **Dora** | F | 28-07-08 | 1 | 26 | 21 |  | Control | 1 |  |
|  |  |  | 2 |  |  |  |  |  |  |
| **Dream** | F | 28-05-08 | 1 |  |  |  |  |  |  |
|  |  |  | 2 | 40 | 29 |  | Control | 5 |  |
| **Ewine** | F | 27-07-09 | 1 | 56 | 1 |  | Control | 4 |  |
|  |  |  | 2 | 10 | 10 |  | Cvs.NC |  |  |
| **Fana** | F | 20-02-10 | 1 |  |  |  |  |  |  |
|  |  |  | 2 | 33 | 127 |  | Cvs.NC |  |  |
| **Felipe** | M | 22-05-10 | 1 |  |  |  |  |  |  |
|  |  |  | 2 |  |  |  |  |  |  |
| **Feya** | F | 05-08-10 | 1 | 15 |  |  |  |  |  |
|  |  |  | 2 | 55 | 17 |  | Control |  |  |
| **Flute** | F | 18-09-10 | 1 |  |  |  |  |  |  |
|  |  |  | 2 |  |  |  |  |  |  |
| **Harlem** | M | 16-07-12 | 1 |  |  |  |  |  |  |
|  |  |  | 2 |  |  |  |  |  |  |
| **Hermine** | F | 05-03-12 | 1 |  |  |  |  |  |  |
|  |  |  | 2 |  |  |  |  |  |  |
| **Lips** | F | 24-11-15 | 1 |  |  |  |  |  |  |
|  |  |  | 2 | 166 | 66 |  | Control |  |  |
| **Lome** | M | 29-08-15 | 1 |  |  |  |  |  |  |
|  |  |  | 2 |  |  |  |  |  |  |
| **Mako** | M | 24-07-16 | 1 | 109 | 34 |  | Cvs.NC |  |  |
|  |  |  | 2 | 99 | 29 |  | Control |  |  |
| **Mali** | F | 08-02-16 | 1 | 43 | 6 |  | Cvs.NC |  |  |
|  |  |  | 2 | 38 | 51 |  | Control | 4 |  |
| **Muse** | F | 21-06-16 | 1 | 2 | 23 |  | Control | 10 |  |
|  |  |  | 2 | 17 | 16 |  | Cvs.NC | 4 |  |
| **Nekke** | F | 24-10-17 | 1 | 53 |  |  |  |  |  |
|  |  |  | 2 |  |  |  |  |  |  |
| **Petoulette** | F | 14-03-99 | 1 |  |  |  |  |  |  |
|  |  |  | 2 |  |  |  |  |  |  |
| **Pipo** | M | 28-08-99 | 1 |  |  |  |  |  |  |
|  |  |  | 2 |  |  |  |  |  |  |
| **Violette** | F | 20-12-04 | 1 | 3 | 1 |  | Cvs.NC | 30 |  |
|  |  |  | 2 | 60 | 40 |  | Control | 23 |  |

**S2 Tables. Stimuli details.** In the tables below you can find the descriptions of the videos which were used as stimuli. The trajectory and duration in ms of object A is shown in orange and of object B in purple. The shown trajectories are in scale. Object A and B always have the same speed. The spatial gap is always ¼ of the distance from the initial position of A to the end position of B, the temporal gap is always 1 second.

**S2A Table.** **Phase 1: L vs. STG, part 1** Videos 1 and 2.

| No. | Event type | Duration (ms) and distance of movements and pause | | | | Total duration (ms) |
| --- | --- | --- | --- | --- | --- | --- |
| 1 | L |  |  | 0 | | 1600 |
|  |  | 1200 | | | 400 |  |
| 2 | STG |  | 1000 |  | | 1600 |
|  |  | 200 |  | 400 | |  |

**S2B Table. Phase 1: L vs. STG, part 2** Videos 1, 2, 3 and 4.

| No. | Event type | Duration (ms) and distance of movements and pause | | | | | Total duration (ms) |
| --- | --- | --- | --- | --- | --- | --- | --- |
| 3 | L | 0 | | |  |  | 800 |
|  |  | 200 | 600 | | | |  |
| 4 | STG |  | |  | 1000 |  | 2200 |
|  |  | 800 | | |  | 400 |  |

**S2C Table. Phase 2: Generalisation 1** Videos 1, 2, 3 and 4 as baseline trials and 5, 6, 7 and 8 as probe trials.

| No. | Event type | Duration (ms) and distance of movements and pause | | | | | | | | | | Total duration (ms) |
| --- | --- | --- | --- | --- | --- | --- | --- | --- | --- | --- | --- | --- |
| 5 | L |  | | 0 | | | | | | | | 1600 |
|  |  | 1067 | | | | | | 533 | | | |  |
| 6 | STG |  | | | | | 1000 | | |  | | 2200 |
|  |  | 900 | | | |  | | | | | 300 |  |
| 7 | L |  | | | | | 0 | | | | | 1200 |
|  |  | 900 | | | | | | | 300 | | |  |
| 8 | STG |  |  | | | | 1000 | | |  | | 1450 |
|  |  | 300 | | |  | | | | 150 | | |  |

**S2D Table: Phase 3: L/STG vs. rest** Videos 1, 2, 3, 4, 9 and 10.

| No. | Event type | Duration (ms) and distance of movements and pause | | | | Total duration (ms) |
| --- | --- | --- | --- | --- | --- | --- |
| 9 | SG |  | | 0 |  | 1200 |
|  |  | 800 |  | | 400 |  |
| 10 | TG |  | | 1000 | | 2600 |
|  |  | 1200 | | | 400 |  |

**S2E Table: Phase 4: Generalisation 2** Videos 1, 2, 3, 4, 9 and 10 as baseline trials and 11, 12, 13 and 14 as probe trials.

| No. | Event type | Duration (ms) and distance of movements and pause | | | | | | | | | Total duration (ms) |
| --- | --- | --- | --- | --- | --- | --- | --- | --- | --- | --- | --- |
| 11 | SG |  | | |  | | | 0 | |  | 1500 |
|  |  | 1000 | | | | |  | | | 500 |  |
| 12 | SG | 0 | | | | | |  | |  | 900 |
|  |  | 225 |  | | | 675 | | | | |  |
| 13 | TG |  | | |  | | | 1000 | | | 2000 |
|  |  | 750 | | | | | | | | 250 |  |
| 14 | TG | 1000 | | | | | | |  | | 1500 |
|  |  | 167 | | 333 | | | | | | |  |

**S3 Table. Categorisation strategies.** We specifically chose the videos to control for irrelevant cues that could lead to correct classification. The following table summarises the cues which can be used for successful categorisation and the cues that can be ruled out because they are controlled for in the videos. The videos which make sure that the cues are ruled out are mentioned between the brackets (see numbered videos in S2 Tables).

|  | **Cues which can be used for successful categorisation** | **Ruled out cues** |
| --- | --- | --- |
| Phase 1:  L vs. STG,  part 1 | - Causality  - Presence / absence of gap (spatial and temporal)  - Presence / absence of spatial gap  - Presence / absence of temporal gap  - Speed  - Movement duration object A  - Distance trajectory object A  - Distance trajectory object B  - Initial position of object B  - End position of object A  - Ratio of movement duration object A to B | - Total duration (1,2)  - Duration before object B starts moving (1,2)  - Movement duration object B (1,2) |
| Phase 1:  L vs. STG,  part 2 | - Causality  - Presence / absence of gap (spatial and temporal)  - Presence / absence of spatial gap  - Presence / absence of temporal gap  - Ratio of movement duration object A to B | +  - Speed (1,4 & 2,3)  - Movement duration object A (2,3)  - Distance trajectory object A (2,3)  - Movement duration object B (1,2,4)  - Distance trajectory object B (1,4)  - Initial position object B (1,4)  - End position object A (2,3) |
| Phase 2:  Generalisation 1 | - Causality  - Presence / absence of gap (spatial and temporal)  - Presence / absence of spatial gap  - Presence / absence of temporal gap | +  - Ratio of movement duration object A to B (5,2,4 & 6,1,3) |
| Phase 3:  L/STG vs. rest | - Causality  - Presence / absence of gap (spatial and temporal) | +  - Presence / absence of spatial gap  - Presence / absence of temporal gap |
| Phase 4:  Generalisation 2 | - Causality  - Presence / absence of gap (spatial and temporal) |  |
